# Medical educators' experiences on medically unexplained symptoms and intercultural communication—an expert focus group study

**DOI:** 10.1186/s12909-022-03275-0

**Published:** 2022-04-23

**Authors:** Viola Sallay, Tamás Martos, Lilla Lucza, Anne Weiland, Karen M. Stegers‐Jager, Peter Vermeir, An Noelle Margareta Mariman, Márta Csabai

**Affiliations:** 1 Institute of Psychology, University of Szeged, Egyetem u. 2, 6722 Szeged, Hungary; 2 Doctoral School of Education, University of Szeged, Szeged, Hungary; 3 Department for Internal Medicine & General Practice, Erasmus MC University Medical Center, Rotterdam, The Netherlands; 4 Erasmus MC, Rotterdam, The Netherlands; 5 Faculty of Medicine and Healthcare sciences, Ghent University, Ghent, Belgium; 6 Ghent University Hospital, Ghent, Belgium; 7 Ghent University Hospital, Center for Integrative Medicine, Ghent, Belgium

**Keywords:** Medically unexplained symptoms, Focus group study, Qualitative analysis, Medical specialists, Medical education, Training development

## Abstract

**Background:**

Medically unexplained symptoms (MUS) are highly prevalent and remain challenging in healthcare and medical education, along with the increase in the importance of intercultural issues regarding MUS. However, less is known about the challenges of professionally addressing patients with MUS in the interprofessional and intercultural contexts. Thus, the present study aims to provide the first exploration of the experiences of medical specialists regarding treating MUS in intercultural contexts and inputs for training development on the intercultural aspects of MUS.

**Methods:**

Three focus groups (total *n* = 13) consisting of medical specialists from a Hungarian university who were teaching at the medical faculty in intercultural settings and also worked for the university health services were interviewed. The topics covered the participants' personal experiences on addressing MUS and the challenges of intercultural communication and the intercultural educational context. Thematic analysis was used to yield a qualitative account of the interviews as guided by the research questions.

**Results:**

Representing the different aspects of medical specialists, the study identified three main themes in the experiences of medical specialists, namely, 1) the need to adapt to the personal world of patients and search for common frames to understand MUS, 2) the need to discover methods for adapting to cultural differences and 3) the need to enhance the interprofessional coordination of knowledge and practices.

**Conclusions:**

The results are in line with the distinct conclusions of previous studies. Moreover, an integrated educational program on the intercultural aspects of MUS may address the main themes separately and, subsequently, support their integration. Therefore, the study discusses the manner in which an integrated educational program on the intercultural aspects of MUS may address the needs recognized in these aspects.

## Background

The term ‘medically unexplained symptoms’ (MUS) refers to various somatic complaints and syndromes (e.g., chronic fatigue, chronic pain, irritable bowel syndrome, and fibromyalgia) without somatic and/or psychiatric diagnosis sufficiently explaining the symptoms [1, 2]. Other definitions of MUS emphasize the lack of identified physiological causes of symptoms. In addition, debates continue about the theoretical and practical adequacy of the term itself [3]. According to these controversies, MUS might be regarded as a diverse set of symptoms some of which will subsequently become diagnosable and treatable disorders united only by the current lack of medical explanation. However, regardless of the exact definition, MUS are highly prevalent and remain challenging for healthcare professionals and patients [2, 4–6], the healthcare system [7] and medical education [8, 9].

Recent empirical research identified several aspects of the complexities of MUS-related challenges in professional practice. First, qualitative studies provided in-depth reports of several problems that physicians encounter when faced with patients with MUS. For example, doctors report feelings of a low level of competence and high frustration, shame, and helplessness when they have the personal sense that MUS-related consultations are becoming disordered and unhelpful [10]. Frequently, they may feel untrained to handle the emotional demands of the consultations, leading to feelings of disappointment and mental exhaustion; moreover, professionals may find it difficult to explain MUS conditions satisfactorily in terms of current medical theory [11]. Consequently, qualitative investigations regularly reported negative experiences and crises pertaining to professional identity [5, 12]. In addition, the existing evidence indicates that despite certain similarities in MUS-related representations among the laypeople and medical professionals, which are mostly conveyed through publicly available health-related information, communication between doctors and patients may continue to pose mutual challenges because of differences in the underlying knowledge structure and endorsed therapeutic solutions [13]. Nevertheless, the literature provides helpful strategies for MUS management where appropriate communication routines [14], a supportive therapeutic relationship [15], and availability of professional guidelines [16] play crucial roles.

Second, the research on interprofessional cooperation in relation to MUS is largely missing. For example, it is unknown how doctors, nurses, and health psychologists can construct a common and/or shared explanatory framework for MUS [12]. Interdisciplinary training and collaboration could be the first step in setting the frames of effective teamwork [17, 18]. The results indicate the importance of incorporating communication and consultation techniques in the training of future doctors and healthcare professionals [5, 19].

Finally, with recent societal changes (e.g., international mobility and migration), intercultural consultations are not only increasingly present in healthcare [20] but also frequently demanding for patients and doctors regardless of symptomatology [21–23]. MUS-related communication may indicate an especially complex endeavor in these intercultural settings. A recent review found that MUS patients, especially those who belong to ethnic minorities, often report experiences of dissatisfaction and misunderstandings. On the other hand, healthcare providers with diverse levels of practice (i.e., from undergraduate trainees to senior physicians) found approaching cultural differences challenging (e.g., diverging concepts of illness perceptions and healthcare-related expectations between patients and doctors), and they tended to feel helpless [24]. Consequently, the significance of intercultural communication skills to the quality of MUS management is growing [17, 25, 26]. However, medical specialists rarely receive systematic training to acquire relevant skills during their education, although experts argued for raising the level of intercultural competencies through curriculum development [27, 28].

### The present study

In facing the known challenges to professional conduct towards patients with MUS and adjusting to these challenges from the interprofessional and intercultural contexts, the need for MUS-related medical training becomes evident [28]. Nevertheless, recent research demonstrated that such training is rare [29]. Moreover, as previously discussed, professional conduct in relation to MUS requires careful consideration of the actual socioecological context of the patient and the treatment. This point highlights the importance of including the cultural/intercultural aspects of care and relevant skills for handling such aspects in the process of training [23, 27, 30]. In summary, medical specialists involved in medical education may face several challenges in teaching about MUS and intercultural issues, partly due to the lack of relevant training programs and curricula.

The present study aims to provide the first exploration of the experiences of medical specialists involved in medical education regarding MUS, with a special focus on intercultural contexts. Moreover, it was designed to provide input for training development on the intercultural aspects of MUS at the University of Szeged. The study was part of the Medical Education on Medically Unexplained Symptoms and Intercultural Communication Erasmus + Strategic Partnership Program (MUSIC) [31] led by the universities of Rotterdam (the Netherlands), Ghent (Belgium), and Szeged (Hungary). Therefore, the objectives were in line with those of the MUSIC project.

To meet the goals of the MUSIC program, we conducted an exploratory qualitative study based on focus group interviews where medical specialists working at the medical faculty of the University of Szeged as teachers and clinicians and with experience in the intercultural context of the university were involved. Focus groups of medical experts proved to be a fruitful way of data generation on the highly complex issues of MUS [32–36]; however, none of these studies applied interprofessional groups. Our study’s interprofessional focus group interviews and subsequent analyses were organized according to the following research questions, aiming to provide inputs for training development.

1. How do medical specialists involved in medical education construct the meaning and implications of MUS?
2. What are the challenges and best practices associated with MUS-related practice in health services in same-culture/intercultural situations?

## Methods

### Sample

The context of the study is a large state university, namely, the University of Szeged, in southern Hungary, which has a long tradition in the training of medical doctors and professionals and providing the same degrees in English- and German-speaking curricula. Moreover, in the frame of MUSIC, a training curriculum development subprogram is being implemented at the University of Szeged that aims for the advanced inclusion of MUS-related topics in medical education. Medical specialists who were actively employed at the University of Szeged were purposefully sampled and qualified under each of the following criteria. First, they worked for the university health services and were teaching at the medical faculty at least part-time. Second, they worked in intercultural settings involving visits or lectures with foreign students. Third, their work was related to MUS either in the university health services or as lecturers at the medical faculty.^1^

During sampling, purposive snowball methodology was used to identify the university’s medical specialists who met the inclusion criteria. In summary, 16 medical specialists were identified through personal networks or invited by the research team, out of which 13 agreed to participate. The reasons for rejection were mainly organizational: lack of time for participation or inappropriate time schedule for the focus groups. The participants were grouped into three according to schedule, and they gave their written informed consent prior to participation in the focus groups. The first focus group consisted of four medical specialists from psychiatry, neurology, and emergency care. They were employees at the medical faculty. The second focus group consisted of one lecturer from the medical faculty, one medical specialist in neurosurgery, two general practitioners (GPs), and one health psychologist from the student counseling service. The third focus group consisted of two GPs lecturing at the medical faculty and two psychologists, one working at the international student service and the other lecturing health psychology (See Table 1 for further details). All participants were of Hungarian nationality, and none of them belonged to an ethnic minority. While the moderator, the first author of the study, and interview participants were employees of the same university, they did not belong to the same faculty. Neither were they each other’s close colleagues or dependents in the work hierarchy.

**Table 1:** Demographics of the participants

Focus groups	1	2	3
Number of participants	4	5	4
Age range (years)	32–69	40–62	29–56
Gender			
Male	2	2	2
Female	2	3	2
Professional background	Medical specialists from psychiatry, neurology, and emergency care	One lecturer from the medical faculty One medical specialist in neurosurgery Two GPs One health psychologist	Two GPs Two health psychologists

### Characteristics of the focus group processes

The first author (a health psychology researcher) moderated the focus group (FG) interviews using a semi-structured interview guide constructed during a collaborative process with the international research team. Interview questions addressed the participants' personal experiences on (1) challenges and good practices related to experiences with MUS (2) dealing with specific situations where MUS-related topics were treated in an intercultural context (Table 2). During the interviews, the moderator refrained from providing formal descriptions on MUS and intercultural communication to facilitate the elaboration of individual experiences and group-level understanding. The interview questions were used to guide focus group processes. Still, the moderator provided room for the discussion of emerging topics and aspects. The tone of each FG was open and collaborative, providing rich data on the subject. Interviews lasted for 90–120 min and were video-recorded. Verbatim transcriptions were produced from the discussions.

**Table 2:** Themes and questions used as guidelines in focus groups

**1. Experiences with’medically unexplained symptoms’ (MUS)**
What do you mean by the term’medically unexplained symptoms’? In what situations do you use this term?
What reasons / precedents / background can you identify behind the appearance of MUS in specific cases? Can you recall a concrete example?
What protocol do you follow in the treatment of MUS? To whom do you refer patients with MUS when a referral is needed? What specialists have to be involved?
What difficulties do you face when interacting with patients with MUS? What represents the most severe difficulty in those dialogues? Can you recall a concrete example?
**2. Communication on MUS in intercultural situations**
What professional encounters / consultations do you consider as being’intercultural’?
What gives you self-confidence in consultations where intercultural communication is needed? What makes you unsure in those situations?
Can you recall concrete examples when you interacted with culturally different patients on MUS? What is different in these situations compared to communication on MUS with patients from your culture?
What is the biggest challenge in communicating with culturally different patients on MUS?
What are your communication strategies in these situations (interactions with culturally different patients on MUS)? Can you recall an example when you managed the consultation successfully on MUS with culturally different patients?

### Data analysis

Inductive thematic analysis [37] was used to yield a qualitative account of the interviews as guided by the research questions. Similar to a study on the views of clinical practitioners on MUS [15], the current study underwent a six-phase inductive process of data familiarization, initial code generation, theme articulation, theme review, theme definition, and narrative reporting. We applied thematic analysis within the frames of a qualitative methodological paradigm, together with an inductive approach to coding and an interpretivist and constructivist approach to analysis [38, 39]. The coding process is assumed to provide theoretical freedom and a practical tool for handling complex meanings in the texts (c.f., 37). First, data were analyzed and coded by the first author. A second independent researcher (second author) analyzed selected parts of the interviews. In a consecutive sequence of discussions between the coders, independently generated codes were revised. The experiences of the revision process were used to finalize the coding scheme used by the first author. A predominantly semantic approach to the data was used (focusing on the explicit level of the text). At the same time, the constructive nature of the data generation process (the participants' formulation of their utterances) and the role of the group interactions were acknowledged. In line with our interpretivist approach, we elaborated a two-level system of themes and sub-themes relying on the centrality of the meaning of the codes and quotes [38]. We developed the three central themes based on the integration of the system of meanings the focus group participants assigned to their experiences. To ensure the integrity of the process, we analyzed the interviews in Hungarian and only codes, and excerpts were translated. The first and the second author discussed the preliminary themes and the translated quotes with the international research team (authors of the paper) and then formulated the definitive system of themes.

## Results

Three main themes and 13 sub-themes were identified (Table 3), which condense professional experiences on how medical specialists constructed MUS-related phenomena and the challenges of dealing with MUS in health services, education and intercultural context.

**Table 3:** Themes from thematic analysis

Theme 1: Adaptation to the personal world of patients
1. 1. Listening to fears and concerns
1. 2. Validating emotions and perceptions
1. 3. Shared understanding of symptoms
1. 4. Communicating ‘lack of knowledge’ and referral
Theme 2: Adaptation to cultural differences
2. 1. Culturally sensitive doctor–patient relationship
2. 2. Openness without fear
2. 3. Culture-specific meanings of symptoms
2. 4. Interculturalism in education
Theme 3: Need for interprofessional coordination
3. 1. Need for consensus view on MUS
3. 2. Coordination between the physical and mental health professionals
3. 3. Need for MUS-specific protocols
3. 4. Coordination in education

### Theme 1: Adaptation to the personal world of patients

The first main theme focuses on the processes that, in the experience of professionals, are most supportive in building and maintaining the doctor-patient relationship. The practitioners’ primary means of reaching this goal is to adapt to the patient's emotional and cognitive reality concerning their MUS-related experiences.

#### Listening to fears and concerns

The three groups highlighted that efficient communication from the physician's part involved listening to the beliefs, views about symptoms, and expression of fears of patients with MUS.

‘I have to explore the reasons why she visited me. 'Cause if I learn her language [i.e., the way she expresses herself], she will tell her fears’. (FG2; subsequent numbers denote the referred focus group)

Conversation without anger and carefully listening to the problems of patients were experienced as good strategies. In this manner, in FG1, all group members supported the claim that physicians did not need to focus on quick answers. They would instead let the patient explore their complaints. According to a GP (FG2), the ability to discuss patients’ emotions and frustrations was beneficial for physicians. He had extensive experience asking MUS patients about the emotions they manifested and the social context behind them. The others in the group approved and validated his approach.

#### Validating emotions and perceptions

One GP emphasized that they seriously considered the description of symptoms, feelings, and solutions of patients, even when deemed absurd. All groups agreed that telling patients, ‘there is no problem’ equated an insult to most patients with MUS.

‘You must not say that she is all right. And in fact, I don't say that either because this is an insult on her. This is a negation of her problem’. (FG2)

Other interviewees complemented the validation of the patient's feelings and notions by transmitting that doctors cared about their health. They deemed this feedback successful in making patients feel comfortable.

#### Shared understanding of symptoms

Understanding the family background and social context of the patient turned out to be an important topic in FG3. Unfolding these themes could promote shared understanding when patients talked about other symptoms apart from their original reason for the visit. Therefore, one GP initiated conversations on intimate or taboo issues (i.e., sex, money, position, and fears) in the case of MUS. Their practice promoted a shared understanding of symptoms in most cases.

Two groups highlighted the role of psychologists in the mutual understanding of symptoms. The health psychologist in the third focus group focused on involving patients in the process of understanding the diagnosis rather than presenting it to them (FG3). The health psychologist in FG2 cited a case in which the symptom (inflammation) that appeared during consultation was related to an actual family conflict stemming from the patient's childhood. In their opinion, a psychologist's goal was to attain a deeper understanding of the patient’s experiences while the patient can increase her awareness about the connection between mind and body.

#### Communicating ‘lack of knowledge’ and referral

Apart from understanding a patient's symptoms and fears, multiple methods of referral were mentioned as part of physician’s routine. As physicians stated, one of the most frequent strategies was referring the patient to systematic physical tests (i.e., laboratory tests and X-ray). ‘Then there's a mental guidance, and there's “a test”’ (FG3). Aside from referral, physicians had to communicate the uncertainty of diagnosis and ‘lack of knowledge’ in the case of MUS. Physicians were frequently concerned with only probabilities at hand. Enabling the patient to understand that physicians could not pinpoint the cause of a blackout and only assumptions could be made was difficult for them. Physicians used different phrasings (e. g. ‘may be caused by internal or external factors’) (FG1). At the same time, the physicians expressed that the role of physicians forbade the presence of uncertainty despite the lack of explicit knowledge on the diagnosis. Moreover, this is how patients could accept the opinion of the physician (FG1).

‘I think it's not OK when the patient sees the physician being uncertain or unsure. So, it shouldn't be revealed, I suppose, or at least I'm trying not to reveal it but to show a calm, determined action’.

### Theme 2: Adaptation to cultural differences

In addition to the focus on personal views and explanations, participants also provided several examples of the processes where understanding and handling cultural aspects of consultations and symptoms played a distinct role. This was true for medical care situations and for experiences of intercultural relevance in medical training.

#### Culturally sensitive doctor–patient relationship

To increase adherence, physicians selected family members with whom they could communicate most effectively. With Roma patients, addressing family members (e.g., grandmother or a ‘voivode’, the leader of the extended family) was found crucial (FG1). The physicians with different professional backgrounds elaborated the cultural aspects of communicating the results of MUS diagnosis to patients (FG1).

‘A.: Maybe about them, about the Roma people, the problem is that it's even more difficult for them to accept that: when I can't answer that question … because the question, of course, is always “what's my problem?” That's the question always’.

MOD.: Yes.

A.: And to them, when I start to say this … (slight chuckle) or so to say, start to yak … yak that the MR was negative…, that everything's fine, the MR hasn't shown anything serious…, no tumor, not this, not that … I can only say what was negative, right, what isn't there. OK, but what then? So … “what's my problem?” And, of course, they ask this a million times. And after all, they are right, because they are visiting the doctor (slight chuckle) to find out what's their problem. So … it's harder for them to accept this, but I, I don't think that this depends on the culture, instead, it depends on the person; I mean there are also other than Roma people for whom it's hard to accept …, so it's not necessarily the culture. Of course, it surely plays a role in it …

B.: Well, I see this family-centricity. So that, a… he [the patient] is curious, (laughing) because he's going to have to tell the others out there, because he's the head of the family, and then...

A.:Yes. (laughs) That is also true…

B.: ...and now how am I going to tell the others why… what's wrong with him… Well, I tell you... (laughs)

A.: Especially the voivode, because the voivode often appears, the voivode, and then he has to know... ’ (FG1)

Although practicing culturally sensitive communication, physicians learned to change their conventional ways, which required continuous attention. According to a GP, women from the Middle East were always escorted by a man, and therapeutic decisions had to be made with him (FG3).

‘Well, as a GP, one practically always has to pay attention to how they relate to this, namely how I … it affects their adherence whether I communicate with them accordingly to their culture or not. For example, a, a religious, um, Islamic girl …. She is always escorted by a male relative. It was odd initially, but we got used to it. And they only accept my prescription on what to buy, what medicine to take if I speak directly to the male relative. I explain to him why they need those and then it's fine’. (FG3)

#### Openness without fear

To realize a culturally sensitive relationship during doctor-patient meetings, physicians had to accept phenomena that they may not accept as a person. Participants found that, as physicians, showing interest in differing cultural groups while ensuring they did not become part of it was beneficial (FG3).

‘Mm, I'm trying to learn from differing cultures what's different; that's what I'm interested in. I'm glad that as a physician, how to say, I can have a taste of a different culture while not becoming a part of it. OK? I have my choice, and most likely, I will choose my own culture. I was born here and all …, but I chose my culture because that's what I like, that's what feels good. I'm interested in other cultures, and, as a physician, I can fit in. I look around, taste it, and I can come back. And I'm not becoming a part of it’. (FG3)

#### Culture-specific meanings of symptoms

The professionals recognized that in culturally sensitive practice, an open approach to the patients’ experiences would aim to understand the culture-specific meaning of symptoms. For example, in Muslim culture, a broken left hand can lead to obstipation due to the ‘tidy hand–dirty hand’ differentiation.

'This is what Muslims have, that is, the difference between the clean and the dirty hand. So the first step was when it came to eating with the right hand and wiping with the left. And his [the patient’s] left hand was broken. And he complained that there was this obstipation and he couldn't defecate. And then, of course, when I realized, whoops, of course, this is because he can’t wipe, so, so he’s on a forced path, and that, that is... to what depths you have to think that their culture is different.’ (FG3)

Several group members pointed out that the significance of physical symptoms had to be determined with the cultural context and cultural meaning of a patient's situation in mind. To this end, physicians had to switch between cultures every 5 min according to a physician attending international students. For example, where a lone Japanese student in an international student group was typically struggling to raise their problems, Iranian students told theirs immediately (FG3). The general practitioner and the psychologist in FG3 (speakers C and D in the following excerpt) agreed that the same cultural difference in meaning and significance of symptoms appeared when they asked the Japanese and Iranian students about their problems at the doctor's office:

‘C: It's funny, the nurse in my office used to say that if an Iranian boy complains, there's nothing wrong with him, he'll get an appointment, but if a Japanese boy comes in and says he's hurting anywhere, we'll send him right to the emergency room (laughs) because it's sure he's got a big problem…

D: It’s different, for example, in the Far East, it's not an individual society, so that there, for example, it's considered gossip if they talk about themselves in this way.’ (FG3).

In addition, the exploration of a patient's notions could promote the understanding of culturally distinct interpretations. A physician in FG1 reported on how she explores the culturally shared subjective experience behind symptoms without organic cause (a headache):

‘Cultural differences can come to light if I ask them about their supposition. In that case, well … sometimes, even things like … “I know, I've been cursed, so ….” (laughs)’. (FG1)

#### Interculturalism in education

Creating common principles of communication in groups of international medical students was found difficult because of cultural differences. According to a faculty member, Japanese students tended to act distant, which could be interpreted as a lack of empathy for others, whereas African students were struggling to maintain eye contact with female patients (FG2). Another group shared more positive intercultural experiences in medical training. This group agreed that such situations shed light on different worlds, thus enabling students to act ‘without judgment’. To support students’ ability to quit from their own world became a common point among group members (FG3).

‘Medical training should make you capable of not judging. You may have your personal opinion that he is responsible for his illness; but in any case, you need to learn how to overcome your preoccupations and just cure him. This makes you capable to deal with cultural differences too’. (FG3)

### Theme 3: Need for interprofessional coordination

Theme 3, which emerged spontaneously from the focus group discussions without explicit questions, groups together aspects that address the fundamental need to coordinate theories and practices between different professionals. Accordingly, the theme also included the need for MUS coordination in medical education.

#### Need for consensus view on MUS

Searching for experiences and coping with uncertainty characterized recurring moments of interactions in the groups. This was also apparent around the concept of MUS. Only a few health professionals and medical faculty members used exactly the term MUS. For others, the focus group interview was the first forum where they met the exact term (FG1), while the group agreed that the phenomenon pertained to ‘psychosomatics’ or could be viewed as a synonym of other more common terms (i.e., conversion and somatization) (FG1). Eventually, during the group discussion, a young specialist remained doubtful about the genuineness of the term MUS.

‘But, is there such a thing that medically unexplained at all? … I mean, is there a definition for that? 'Cause I might have been watching too much Doctor House probably, and it was always revealed that … some toxicological, I mean, some poisoning was there, heavy metal … or some infection was always revealed’. (FG1)

#### Coordination between the physical and mental health professionals

In certain ways, all groups considered the possibility of coordination between physician, psychiatrist, and psychologist in the care for patients with MUS. However, medical doctors were uncertain about the methods of psychological and psychiatric care in terms of whether the terms shared the same meaning, whether they understood the difference and which one they would propose as a physician (‘I understand the difference, or I hope so that I do, but I, as a physician propose psychiatry’ [FG2]). One of the GPs experienced difficulty in persuading patients to attend even free psychological treatment. However, this form of attendance could be introduced slowly (FG3). To increase the efficiency of referring psychologists, a GP encouraged patients to receive a psychologist's help by stating that the vegetative nervous system is accountable for these symptoms, which can be alleviated by peace of mind (FG2; speaker ‘F’ in the following excerpt). However, another GP (speaker ‘E’) would oblige patients with MUS to undergo psychological treatment.

E.: ‘And um, essentially I was thinking about this, that if you can completely, so if you can trivially say that, that there's, there's um, psychological causes, then why don't I have the right to quasi oblige them to, that this person, that they go to such therapy and don't make demands on healthcare that is already lacking resources. Or, that …

F.: You have the right, you have the right, but it's very difficult’. (FG2)

When feeling helpless, referral to a psychiatrist was considered the ‘last refuge’ for the physician in case of patients with MUS. At the same time, the referring specialist was unaware about what happens with the patient in the psychiatry ward.

‘But we also have, you know, a kind of “last refuge”, the psychiatry (someone laughs). Well, I don't know even after more than 20 years however, what, what a psychiatrist can possibly say. ‘Cause in this kind of cases they are the, the last refuge …’. (FG1)

The psychiatrists, in turn, found MUS as a valuable concept because they understood the physical and mental aspects of diseases as well. However, patients referred to psychiatry due to uncertainty occasionally displayed physical problems (FG1). To this group, the most important conclusion of the interview was that members missed opportunities for interdisciplinary physician–psychiatrist–psychologist meetings. Thus, the current study was filling this gap for them.

#### Need for MUS-specific protocols

The lack of protocols on patients with MUS in either primary or specialized care appeared to be a general problem for all groups. The GP alternately working in Great Britain and Hungary missed clear protocols in Hungary as opposed to that observed during an experience abroad:

‘There was the experience in GB that protocols were for knowing one's rights, so we had to explain that they don't deserve more, that that's their right in this system. So there, they drew the line there, that's the protocol, that way. A, B, C, so … That's their right. And this, this has to be accepted, and they have to accept this, that this system means this. For us, it's looser; we give more to some and less to others. Not because we make a difference, but for … how much one needs to calm down or to accept the thing’. (FG3)

Lack of clear protocols on the treatment process and MUS-related interprofessional coordination also meant that specialists did not have feedback from referred patients. This, in turn, led to a low sense of accomplishment and lack of information: the only (negative) feedback was when patients returned again and again to specialized care (FG1).

#### Coordination in education

A psychologist opined that communication training was essential for preparing medical students to care for patients with MUS: students could learn a holistic approach for patients without focusing on symptoms. Physicians in the group agreed with this notion. Medical students had to spend much more time in a GP’s praxis ‘because that's where real life is’, that is, where they could see how to deal with MUS patients (FG2). Others proposed that self-knowledge and Balint groups should be part of medical education to help students gain self-confidence in patient-physician communication. However, these and other communication training groups were considered few and expensive (FG2).

‘There would be great need in the training of specialists (e.g., emergency) to learn about this. By now, there is no such theme in the specialist exam, they don't even hear about it (FG1). Even more, a resident physician gets warned when a patient education lasts too long. That contradicts to what is being taught about how to communicate MUS symptoms, namely, it is worth spending time with, listening to and informing MUS patients, because this can prevent frequent visits’. (FG1)

The psychiatrist in FG1 pointed out as a general problem that medical education continued to prepare students for biological and acute problems and saving lives, whereas the majority of patients presented with chronic conditions. In addition, positive physician–patient relationships and communication from the beginning were deemed important for healing chronic cases, such as MUS. According to the group’s final opinion, these aspects needed much more focus in standard medical education.

## Discussion

To the best of our knowledge, this qualitative study is the first to explore the MUS-related aspects of medical care, focusing on the experiences of medical specialists involved in medical education and intercultural communication. The study explored these experiences to understand the dual aspects of (1) the meaning of MUS and its implications for the participants’ professional attitudes and identity, and (2) challenges associated with MUS and the corresponding needs and best practices of professionals in university health services and medical training within intercultural and same-culture situations. The results represent the complex challenges of professionals who work with diverse target groups, such as patients with MUS and patients with an intercultural background, medical students (either with a same-culture or an intercultural background), and other health care professionals. Moreover, the focus group discussions also provide insight into how the participants’ practices relate to their MUS-related professional understanding and identity.

Three main themes emerged. First, the focus groups identified specific means for healthcare professionals to adapt to the personal world of patients within intercultural and same-culture settings to reach a shared understanding of MUS and promote an optimal professional relationship. Communication practices included i) listening to fears and concerns and validating the emotions and perceptions of patients, ii) striving to reach a mutual understanding of symptoms and, when required, communicating ‘lack of knowledge’ and iii) referring to other specialists. Similar to the present study, previous qualitative studies cited a struggle for doctors in expanding their approaches across consultation and knowledge bases to meet the challenges of incongruence between dominant disease models and the biopsychosocial reality of patients [5, 36]. In the process of adapting to the personal world of patients, healthcare professionals faced several aspects of communication challenges from the conveyance of a physician's understanding of the reality of the clinical situation [13] during the early discussion of MUS and providing care without presenting MUS as a diagnosis for exclusion [35] to the possibility of psychiatric referrals and the role of the Internet in the management process [15]. In this way, their struggle for effective practice has also shaped their professional attitudes and identity.

Second, the results demonstrated that medical specialists were aware of the need to adapt to cultural differences during visits. However, they struggled to achieve a satisfactory solution for themselves: to build culturally sensitive doctor-patient relationships, approach patients’ personally challenging cultural practices openly and fearlessly, and understand the culture-specific meanings of symptoms and health behaviors. In discouraging and positive ways, specific aspects of intercultural challenges were present in multicultural medical training settings (see Interculturalism in the education sub-theme). The results are in line with scholarly notions on including cultural aspects in healthcare praxis [17, 20, 25, 40–42]. Regarding MUS, a complex culture-focused investigation should include psychological concerns and screening for a history of dysfunctional childhood and symptoms of depression, anxiety and PTSD, and family and cultural background [25]. However, such requirements – along with addressing physical and medical aspects of the symptoms – may place additional burden on medical specialists [33]. The respondents confirmed the challenges of intercultural communication about MUS found in previous studies [14, 16, 17, 41, 42]. At the same time, they were aware that dealing with the intercultural aspects of consultation often required changes in their attitudes.

Coping with challenges was further elaborated in the third theme, where the need for interprofessional coordination became key. Several drawbacks to and necessary developments of coordination were cited. For example, the term MUS remained an ambiguous concept among professionals signifying the lack of common terminology in interprofessional communication. However, developing reliable protocols for patients with MUS and, in a general sense, coordinating training elements in the education of future health professionals might support the uncertainties of coordination between the physical and mental health professionals. The need for interprofessional coordination was an overarching theme in the interviews. Medical specialists confirmed that communication about MUS is crucial for colleagues and multiprofessional teams. Previous studies on MUS also cited the need to work in multidisciplinary primary care teams [12, 17, 18, 43, 44].

The themes demonstrate that medical specialists are active agents in the construction of their professional identity, practice and the corresponding social environment. They strive to develop personal skills, determine viable consultation strategies [32], and make suggestions for the broader systemic aspects of care while also reflecting on their thoughts and feelings. The focus group discussions revealed a great interest in and openness to the topic among the medical specialists, which led to the emergence of several relevant aspects and themes. In this sense, focus group discussions provided in vivo experiences on the viability of a constructive interprofessional exchange on a controversial and challenging subject.

### Implications for medical education

We interpret the results that MUS-related professional functioning in same- and intercultural settings is a multi-layered and complex adaptation process. This process involves several aspects of systemic functioning, such as the interrelated challenges of personal, intercultural, and interprofessional adaptation. Moreover, the adaptation process includes the need for professional development in skills and attitudes. Therefore, the results also show that, since the professional adaptation process is multi-layered and complex, there is an urgent need for related training programs in medical education. Consistent with the objectives of the MUSIC project [31], the themes outline building blocks to an integrated training program with a focus on the multi-layered adaptation process to the challenges of MUS, especially in the intercultural context. We may also note that each of the three main themes contains subthemes that point out more common and trainable issues and subthemes whose educational realization is less evident (see details below). Consequently, an integrated educational program on the intercultural aspects of MUS may address the main themes separately and, subsequently, support their integration.

First, the integrated educational program may involve developing MUS-related personal knowledge and communication skills (c.f., Theme 1). Adequate listening and interpretation skills are common basic expectations towards medical specialists [45]. Results confirm that training elements need to address emotional (e.g., validating emotions) and cognitive aspects (e.g., reaching shared understanding) of the MUS-consultation parallelly. However, we deem the constructive handling of the ‘lack of knowledge’ subtheme the most challenging for education. Medical trainees may find it difficult to accept the uncertainty inherent in MUS consultations [46], and facing MUS may challenge a doctor's role identity [5]. Focus group members also expressed the role inconsistencies between the traditional doctor’s role and MUS-related challenges. Accordingly, training on MUS-related issues can promote professional skills and further development of professional identity, whereas emotionally assuring experiences may play a critical role [47].

Second, personal-level consultation skills can be connected to the development of intercultural sensitivity (c.f., Theme 2), and there were suggestions to include cultural competencies in the medical curricula [48, 49]. While conveying reliable information on cultural differences is essential, intercultural sensitivity may tackle attitudes (i.e., non-judgmental relationships) and emotional aspects [50]. Intercultural sensitivity development goes beyond mere knowledge transfer. In this regard, we consider the theme ‘openness without fear’ especially challenging for training development since it involves personal and professional uncertainty and, thus, questions the traditional medical role model. Therefore, addressing uncertainty management needs to be an inherent part of teaching about cultural issues [51] since it can open pathways toward forming better doctor-patient relationships and a deeper understanding of the patients’ symptoms and experiences.

Finally, results also suggest that the preparation for skillful interprofessional coordination of MUS treatment is necessary (c.f., Theme 3). As presented earlier, the MUS concept is controversial and complex, and the focus group members also expressed ambiguity about it, including the use of terms other than MUS and questioning the viability of the concept. Several previously published suggestions pertained to the development of MUS training elements in the higher education of medical professionals, such as doctors, nurses, and health psychologists [2, 28, 36, 52–54], which may help interprofessional coordination. Nevertheless, studies also pointed out the barriers that the educators of such training had to overcome [55]. One focus group tackled this problem when participants considered medical education too focused on rare and particular diseases and conditions instead of providing practice in everyday medical situations. Moreover, various aspects of MUS-related interprofessional relationships, such as referral systems, protocols, and broader aspects of medical training, might be far beyond the scope of a specific training program. However, support for interprofessional coordination may require further professional reflections on multiple—bio–psycho–social—aspects of MUS and the facilitation of professional exchanges, which, in turn, may lead to further developments at a broader, systemic level of healthcare.

### Strengths and limitations

The study sought a general view and incorporated experiences with MUS from participants with different professional backgrounds. The focus groups represented many forms of professionals, such as medical doctors with different fields of specialization (i.e., GPs, psychiatrists, and neurologists) and health psychologists. The participants experienced medical training and clinical practice in a university with several international students in medical training. Thus, their experiences can be applied to the development of future curricula on MUS-related topics in other same-culture and intercultural settings. However, the relatively low sample size and the involvement of medical professionals from only one university limits the transferability of the results. Participants were homogeneous in their cultural background and represented the primarily Hungarian teaching staff of the university. Therefore, we cannot assume that the participants’ experiences represent those of professionals from other universities, disciplines, clinical settings and cultural backgrounds. Therefore, future studies may replicate the current research process in other university contexts and involve professionals from multiple specialities, such as gynaecology, gastroenterology, cardiology, and nurse practitioners [56]. This line of research can be further extended to cross-culturally designed quantitative studies on the clinical management of MUS. Moreover, the discussions did not focus on syndrome-specific experiences. Instead, the participants shared broad experiences, which may also reflect that the moderator was a health psychology researcher who did not have a medical practice. Finally, although we involved a team of international experts (including psychologists and medical doctors) in the preparation of the study, we did not rely on patient and public involvement processes in the research. Future work should further explore this domain by systematically involving patients with diverse cultural backgrounds in the questioning and giving voice to their experiences with medical students, residents and professionals of university clinics. This may provide direct feedback for the training process and, more generally, ensure reflective practice and theory building.

## Conclusions

Teaching about MUS may lead to challenges for medical educators given that a general consensus on the general model of the MUS is missing, whereas multiple conceptualizations and theoretical models exist only as explanations [40, 57–61]. A MUS-related training should target the typically ambivalent and complex nature of dealing with the phenomenon and the challenges of building an interculturally sensitive doctor-patient relationship with communication about MUS. Moreover, it should address the necessity of developing openness and embracing attitude changes at each level of professional functioning, including self-awareness, intercultural competency, patient focus, identity development, interprofessional cooperation, and systemic changes in training and protocol.

## Data Availability

The video material generated during the current study are not publicly available due to limitations of ethical approval involving the participant data and anonymity. Anonymized interview transcripts (in Hungarian) are available from the corresponding author upon reasonable request.

## Abbreviations

FG: Focus group
GP: General practitioner
MUS: Medically unexplained symptoms
MUSIC: Medical Education on Medically Unexplained Symptoms and Intercultural Communication Erasmus + Strategic Partnership Program

## Footnotes

1 During their medical education curriculum, medical students at the University of Szeged learned about MUS in the framework of medical psychology. Classes in the 4th year of training (http://web.med.u-szeged.hu/magtud/angol_ok/Med_psycho2.pdf). Other classes may contain references to MUS (e.g., general practice in Clinical Module).
